# The Effects of Manufacturing Errors on the Performance of Acoustic Metamaterial Lenses Operating in the MHz Regime

**DOI:** 10.1002/smsc.202400481

**Published:** 2024-12-06

**Authors:** Feng Qin, Jie Zhang, Bruce W. Drinkwater

**Affiliations:** ^1^ Department of Mechanical Engineering University of Bristol University Walk Bristol BS8 1TR UK

**Keywords:** accumulated delay method, acoustical metamaterial lenses, Huygens’ principle, manufacturing errors

## Abstract

This article explores the use of acoustical metamaterials to design lenses in the megahertz frequency range of relevance to applications in nondestructive testing and medical imaging. In particular, the effect of manufacturing errors on their focusing performance is quantified. A rapid method for including manufacturing errors is described and this is used to perform Monte Carlo simulations of wave pressure fields from lenses with manufacturing errors. In this process, the required time delay distribution for a target focal length is calculated and the acoustical lenses with a chosen unit cell type are designed. Manufacturing errors of the unit cells are then added, considering their statistical properties and a large number of realizations. As an example, an acoustical lens with a focal length of 76 mm at a frequency of 1 MHz is designed using three different unit cell types: steel cross unit cell, resin circular void unit cell, and silicone–resin layered unit cell. Finally, the resulting acoustic pressure fields are computed using a Huygens’ principle model to assess the effects of manufacturing errors on lens performance, i.e., the focal length and the size of the focal spot. It is shown that the performance of lenses consisting of silicone–resin layered units is less affected by the manufacturing errors than for lenses constructed with the other unit cells. This study paves the way for selecting a suitable combination of metamaterial unit cell and manufacturing method to enable acceptable lens imaging performance.

## Graphical abstract

Herein, a procedure is proposed for evaluating the impact of manufacturing errors on the performance of acoustic metamaterial lenses during the design process. This procedure employs the accumulated delay method and Huygens’ principle to access the performance variations caused by manufacturing errors. Three types of unit cells are used to investigate the versatility of this procedure.

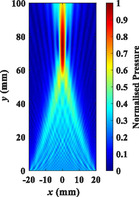

## 1 Introduction

Acoustic metamaterials (AMMs) are artificial composite materials made from at least two materials with different properties and have become an important research topic due to their potential applications in biomedical fields and medical imaging,^[^ ^1^, ^2^, ^3^, ^4^, ^5^, ^6^ ^]^ ultrasonic nondestructive testing,^[^ ^7^, ^8^, ^9^, ^10^, ^11^ ^]^ and for noise reduction.^[^ ^12^, ^13^, ^14^, ^15^ ^]^ These materials can be categorized based on their functionality into following types: acoustic cloaking,^[^ ^16^, ^17^ ^]^ acoustic focusing,^[^ ^18^, ^19^, ^20^, ^21^, ^22^, ^23^ ^]^ acoustic absorbing,^[^ ^24^ ^]^ acoustic manipulating,^[^ ^25^ ^]^ and various metamaterial lenses.^[^ ^26^, ^27^, ^28^ ^]^ Metamaterial unit cells are also classified by their geometries and distribution into 1D and 2D configurations depending on the arrangement of inclusions along one or two directions within the host materials.^[^ ^29^ ^]^

Examples of 1D unit cells include the concentric circular sandwiched‐ring array for underwater focusing^[^ ^30^ ^]^ and the active gradient metamaterial for high‐frequency acoustic sources with broadband response and active self‐focusing underwater.^[^ ^31^ ^]^ Additionally, 2D unit cells include the star‐shaped lattice structure for focusing sound in water,^[^ ^32^ ^]^ the gradient‐index lens with orifice‐type unit cell for focusing sound waves in air,^[^ ^33^ ^]^ and the foam‐like metallic structure for focusing capability in water.^[^ ^34^ ^]^ There are also 2D unit cells with three‐material geometries, such as trapped‐in air structure for beam focusing underwater^[^ ^35^ ^]^ and trapped air structure for subwavelength imaging in water.^[^ ^36^ ^]^

The coupling effects between adjacent unit cells,^[^ ^37^, ^38^ ^]^ along with manufacturing errors, can alter the behavior of AMMs. When using smaller unit cells to reduce coupling issues, manufacturing errors still significantly impact performance. Therefore, precision in fabricating these complex structures is crucial. Fan et al.^[^ ^39^ ^]^ highlight discrepancies between experimental and simulation results due to errors in geometric parameters of the unit cell, while the variation in measured Poisson's ratio of 3D‐printed metamaterials is caused by errors in cylindrical‐shell‐beam units.^[^ ^40^ ^]^ Kennedy et al.^[^ ^41^ ^]^ discuss how different additive manufacturing technologies affect the behavior of the unit cells. Hence, although the effect of manufacturing on metamaterial unit cells has been explored, there is a lack of consideration regarding manufacturing errors in the design processes of acoustical metamaterial application using lenses which consist of many unit cells.

To address this gap, this article aims to develop a procedure for predicting the performance of metamaterial lenses during the design phase, selecting appropriate fabrication methods, and evaluating the optimal metamaterial unit structure.

This article is organized as follows: Section 2 outlines the methodology for acoustical metamaterial lens (AMML) design, including the extraction of effective properties of metamaterial unit cells and the parameter details of metamaterial lenses with various unit cell structures. This section also identifies the geometric changes due to manufacturing errors and their relative significance for different unit cells. Section 3 explains the accumulated delay method (ADM) used to calculate the time delay distribution of multilayered metamaterial lenses, along with a generalized performance evaluation process that considers manufacturing errors. In Section 4, the finite element (FE) method is utilized to validate the performance of metamaterial lenses and ensure they meet design requirements. The effects of the manufacturing errors on lens performance, i.e., the acoustic field and the focusing properties, are then evaluated using a Huygens’ principle model. Section 5 discusses criteria for selecting the manufacturing method and identifies the best unit cell among the three types considered. Finally, the article summarizes the work and presents the key conclusions.

## 2 AMML Design

### 2.1 Lens Design Method

A general method for designing focused AMMLs is the time delay method.^[^ ^18^, ^42^ ^]^ Other methods can be used for lens design in specific scenarios, such as hyperbolic delay profiles, but we implement the time delay method due to its general applicability. In this approach, a plane acoustic wave is incident on and propagates through a flat metamaterial lens. The spatial variation of velocity within the lens then leads to the required spatial distribution of phase/time delays. By designing these time delays such that waves arrive at a desired focal point in‐phase, the lens structure can be constructed using various unit cells. As an example that has relevance to both nondestructive and medical imaging,^[^ ^2^, ^11^ ^]^ we set target requirements of an AMML as follows: an operating frequency of *f* = 1 MHz, a lens width of *D* = 40 mm, a focal length of *y*_f_ = 76 mm and working in water with a wave speed of *c*_0_ = 1500 m s^−1^ and the corresponding wavelength of *λ*_0_ = 1.5 mm.

### 2.2 Unit Cell Design Method

To achieve the required time delay distribution of an AMML, it is essential to determine the effective properties of unit cells with various geometries. This can be performed using methods such as the retrieval method (RM) or the construction of the band structure (CBS) diagrams.^[^ ^18^, ^43^ ^]^ In the RM, a plane wave is assumed to impinge on a unit cell, and the reflection and transmission coefficients are used to extract the refractive index and acoustic impedance of the AMM.^[^ ^18^, ^43^ ^]^ Here, we use the CBS method as it gives direct insight into the wave modes present as well as the location of stop‐bands. Common methods for calculating band structures include the finite‐difference time‐domain method, plane wave expansion method, multiple scattering method, and, as used here, the FE method.^[^ ^44^, ^45^ ^]^

**Figure 1:**
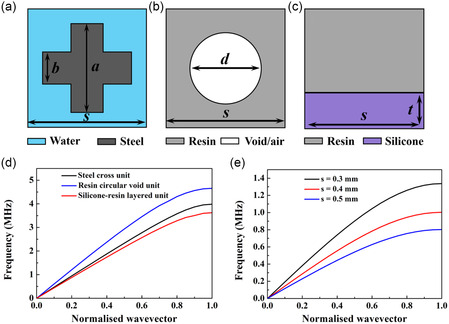
Considered unit cell structures used in water: a) steel cross unit cell, b) resin circular void unit cell, c) silicone–resin layered unit cell, d) the first band structure for various unit cells with a unit size of *s* = 0.15 mm and a geometry ratio of *r* = 0.5, and e) the first band structure of the steel cross unit cell for various unit sizes *s* = 0.3, 0.4, and 0.5 mm at a geometry ratio of *r* = 1. Here, *s* is the unit size of the unit cells and the geometry ratio *r* is defined as r=a/s  for the steel cross unit cells, r=d/s  for the resin circular void unit cells, and r=t/s for the silicone–resin layered unit cells. It is noted that a constant *b = *0.35 *s* is used for the steel cross unit cells.

Three types of unit cell structures working in water, with their geometries shown in **Figure** **1**a–c, are chosen as examples to investigate the effects of manufacturing errors on the performance of metamaterial lenses. The method is general and so is expected to perform effectively when applied to other types of unit cells, such as Helmholtz and spiral structures. As shown in Figure 1a,b, the steel cross and resin circular void unit cells are 2D AMM. In contrast, as shown in Figure 1c, the silicone–resin layered unit is a 1D AMM.

Using the CBS method and considering the requirement of an operating frequency of 1 MHz, the effective properties of all these unit cells were extracted. The maximum unit sizes for the steel cross, resin circular void, and silicone–resin layered units are limited by the presence of stop‐bands in the band structure, as demonstrated in Figure 1e for the steel cross unit. These maximum sizes are calculated to be 0.4, 0.27, and 0.49 mm, respectively, as illustrated in **Table** **1**. It should be noted that, for each unit cell structure, two unit size cases are explored: one is the maximum unit size such that the unit cell is operating just below the first stop‐band at the operating frequency, listed in Table 1; the other is a smaller unit size of *s* = 0.15 mm which meets the requirement for the homogeneous regime, i.e., *s* ≪ *λ*_0_.

**Table 1:** Maximum unit size of different unit cells under operating frequency of 1 MHz.

	Steel cross unit	Resin circular void unit	Silicone–resin layered unit
Max unit size *s* [mm]	0.4 (*λ*_0_/3.75)	0.27 (*λ*_0_/5.56)	0.49 *(λ*_0_/3.06)

**Figure 2:**
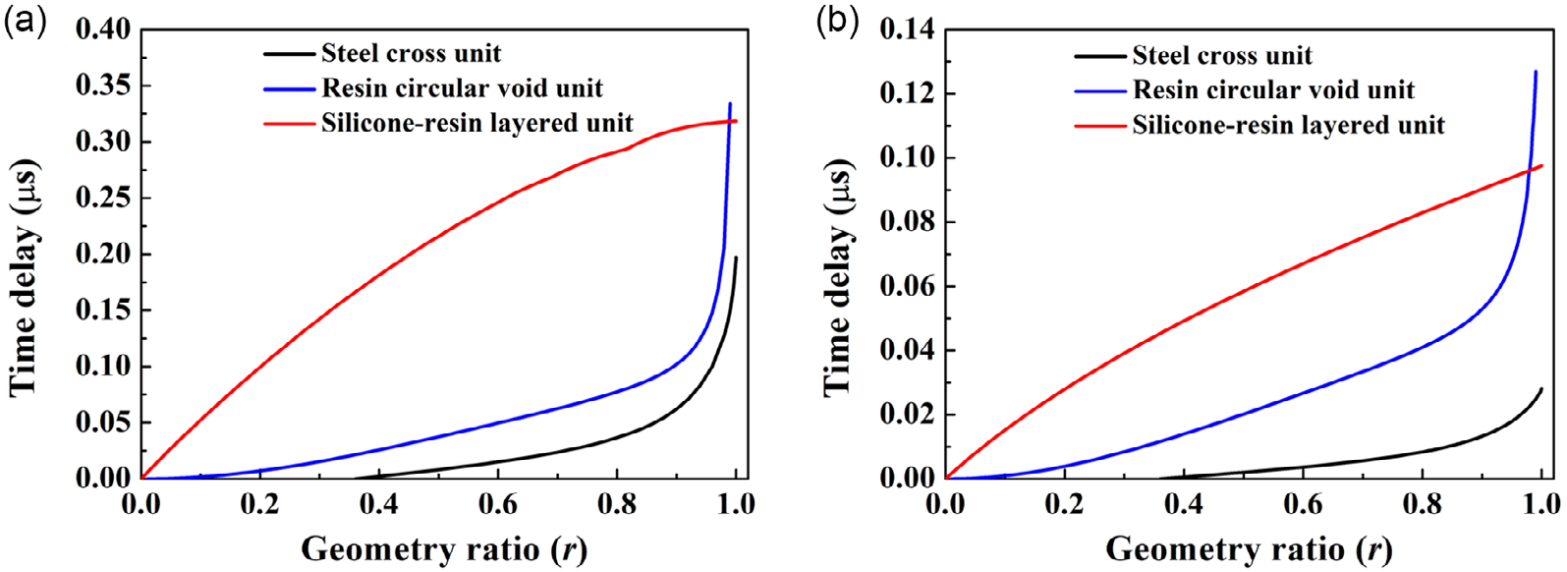
The time delay of a single unit cell as a function of unit geometry ratio for various unit structures including: a) larger unit cells with the steel cross unit with a unit size of *s* = 0.4 mm, the resin circular void unit with a unit size of *s* = 0.27 mm, and the silicone–resin layered unit with a unit size of *s* = 0.49 mm and b) smaller unit cells with all unit types with the size of *s* = 0.15 mm.

From **Figure** **2**a, it is evident that the silicone–resin layered unit cells exhibit a gradually increasing time delay with geometry ratio across the entire range of geometry ratio. In contrast, the other unit cells show a rapidly increasing time delay when the geometry ratio exceeds 0.95. In this regime, small fluctuations of the effective properties of unit cells could lead to significant performance variation of the metamaterial lenses. Hence, in subsequent designs, the maximum geometry ratio for the silicone–resin layered unit cell is 1, while for the steel cross and resin circular void unit cells, it is 0.95.

Using the results shown in Figure 2a,b and following the steps outlined in ref. [18], the details of the designed AMMLs are listed in **Table** **2**. First, the desired time delay distribution of the AMML for a target focal length is determined using the time delay method. The lens thickness and unit cell dimensions can then be found using the data in Figure 2a,b and the ADM (see Section 3 for more detail). From Table 2, it can be seen that the silicone–resin layered unit cells lead to the thinnest lens among these three types of unit cells.

**Table 2:** Details of metamaterial lenses with steel cross, resin circular void, and silicone–resin layered unit cells.

Unit cell structure	Unit size *s* [mm]	Frequency *f* [MHz]	Focal length *y*_f_ [mm]	Thickness *l* [mm]	Width *D* [mm]
Steel cross unit	0.4	1	76	7.6	40
	0.15			14.4	39.9
Resin circular void unit	0.27	1	76	3.51	39.96
	0.15			3.9	39.9
Silicone–resin layered unit	0.49	1	76	2.94	40.18
	0.15			2.7	39.9

### 2.3 The Performance of Unit Cells with Manufacturing Errors

**Figure 3:**
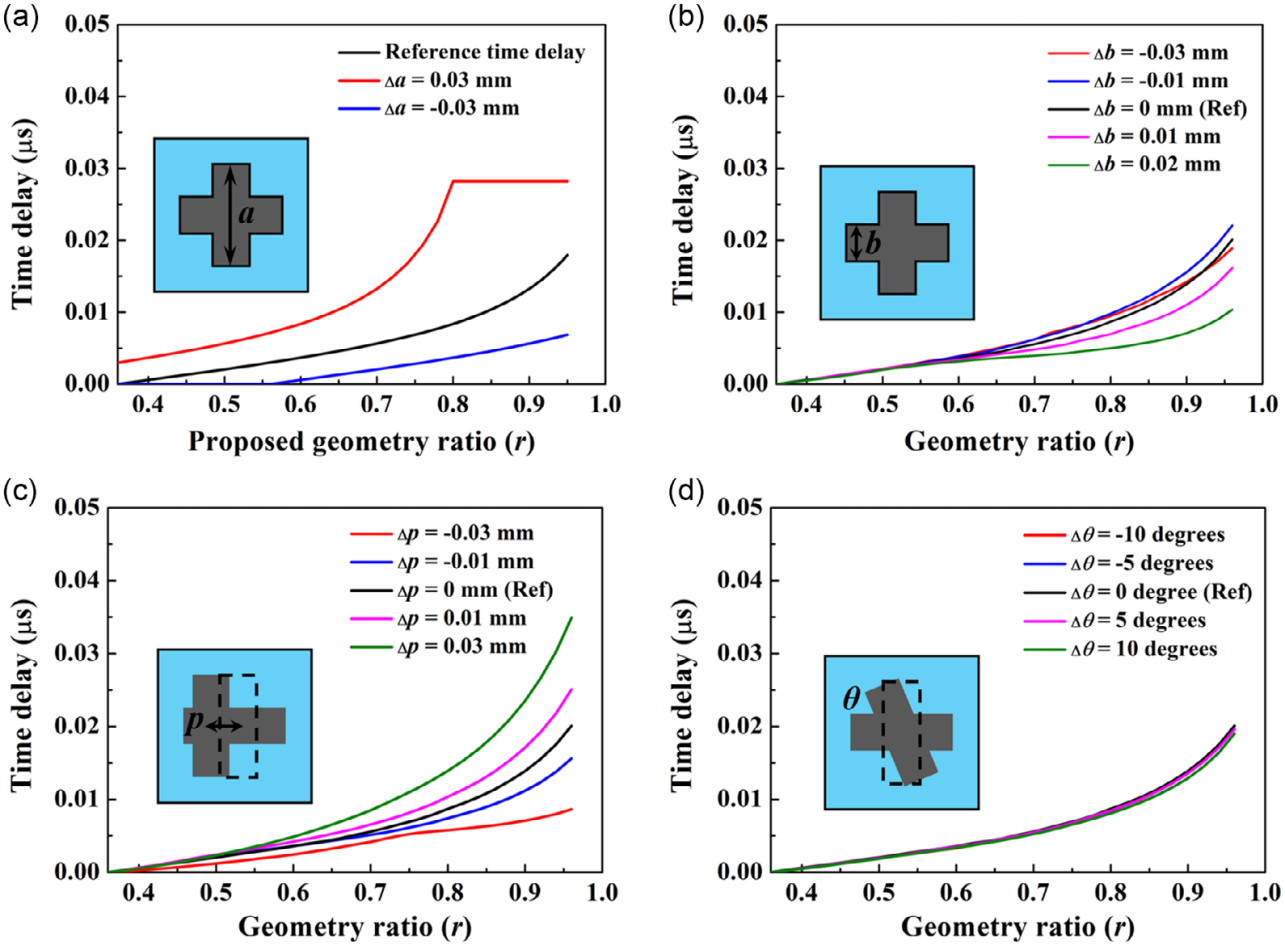
Time delay of a single steel cross unit cell as a function of *r* with considering a manufacturing error from: a) cross height and width, *a*; b) cross thickness, *b*; c) positional, *p*; and d) shape angle, *θ*. The reference unit cell has a size of *s* = 0.15 mm.

AMMLs can be manufactured using a number of approaches and 3D printing is particularly popular.^[^ ^1^, ^18^, ^22^ ^]^ Inevitably every manufacturing process results in manufacturing errors, such as dimensional errors, positional errors, and shape errors. **Figure** **3**a–d shows the variation of time delay from the steel cross unit cell with manufacturing errors from the cross height and width, *a*, cross thickness, *b*, center position, *p*, and cross shape angle, *θ*, respectively. It is shown that the cross thickness and shape errors have relatively small effects on the effective properties of the steel cross unit cell. The variation range of time delay with cross height error is larger than that of center positional error. For instance, the percentage fluctuation in time delay variation relative to the reference time delay at a geometry ratio *r* = 0.7 is 198.76% for cross height error (Δ*a*), 41.73% for cross thickness error (Δ*b*), 77.7% for center position error (Δ*p*), and 4.5% for shape angle error (Δ*θ*). Hence, the dominant manufacturing error for the steel cross unit cell is the cross height error, Δ*a*.

**Figure 4:**
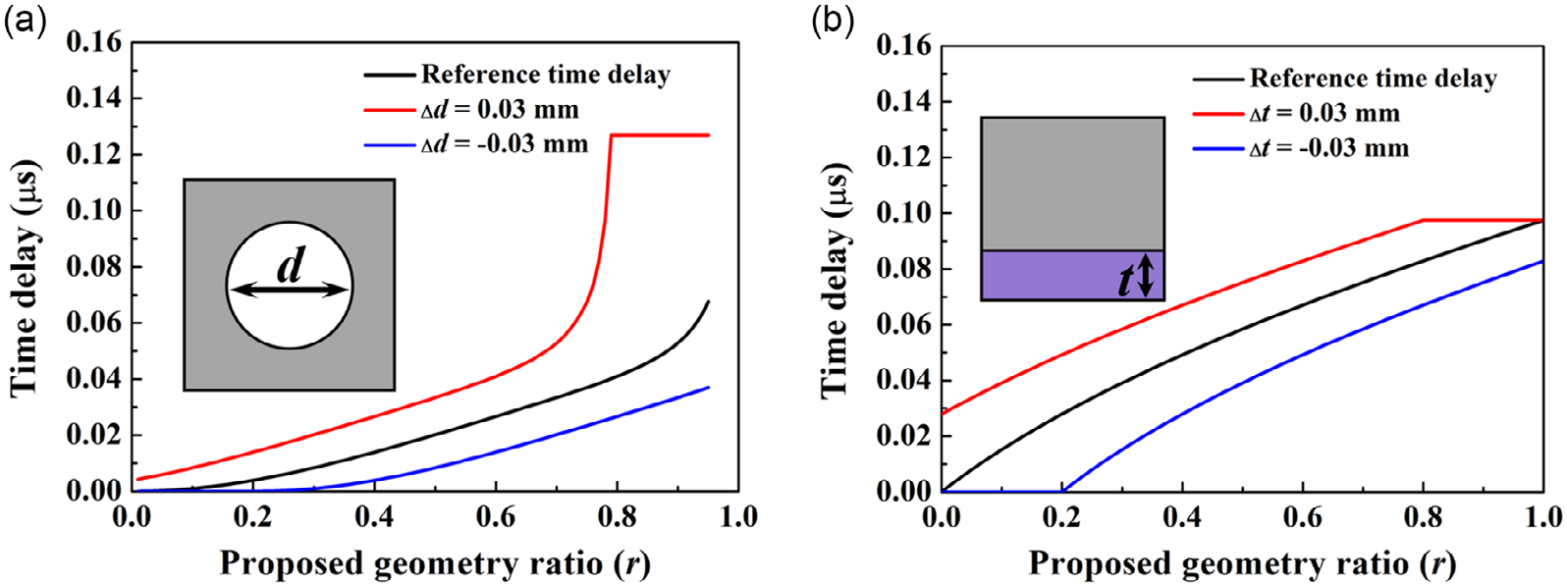
Time delay as a function of *r* with considering a manufacturing error from: a) diameter of void zone, *d*, in resin circular void unit cells and b) silicone thickness, *t*, in silicone–resin layered unit cells. The reference unit cell has a size of *s* = 0.15 mm.

For each type of unit cell, it is observed that one of the manufacturing errors has a significantly greater effect than the other and we term this the dominant manufacturing error. It is crucial to identify the dominant manufacturing error among all these types to validate their effects on lens performance through a practical example. By analyzing the resin circular void and silicone–resin layered unit cells, their dominant manufacturing errors were found to be from the diameter of the circular void zone, *d*, and the silicone thickness, *t*, respectively. The details about the variation in time delay of these unit cells are shown in **Figure** **4**a,b.

## 3 ADM

**Figure 5:**
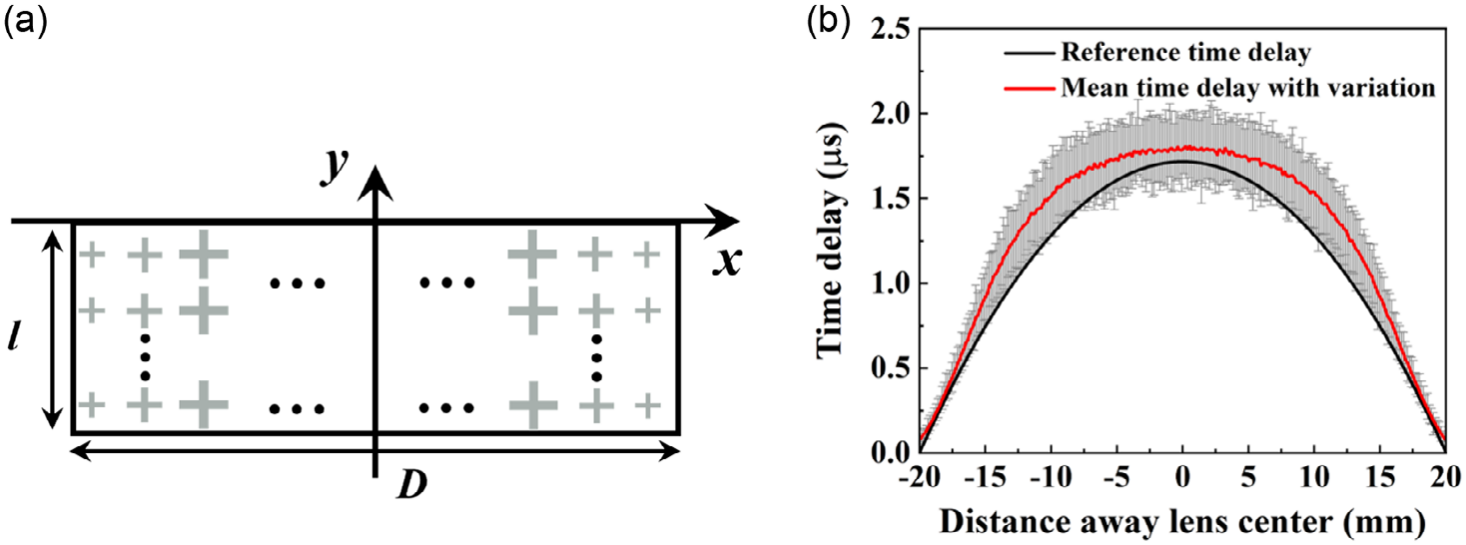
a) The schematic diagram of an AMML with steel cross unit cells, where *D* represents the lens width and *l* denotes the lens thickness, and b) the resulted overall time delay variations determined by comparing the time delays of 100 realizations of various *a* with standard deviation *σ* = 0.01 mm with the reference time delay.

As shown in **Figure** **5**a, the overall time delay distribution of a portion of the lens can be calculated as the sum of the delay due to a number of unit cells, termed the ADM expressed as

(1) $$\tau =\sum_{i=1}^{i=M}\tau_{i}$$

where *τ* is the overall time delay distribution of a lens, $\tau_{i}$ represents the time delay of the *i*th layer of a lens, and *M* is the number of layers. As described in the previous section, we use CBS diagrams found by FE analysis to extract the $\tau_{i}$ and then Equation (1) to find the delay for the multilayer lens structure.

When 3D printing an AMML, each unit cell is printed individually. It is assumed that each individual manufacturing error of a unit cell obeys a normal distribution with zero mean and a specific standard deviation of *σ*. As a demonstration example, the black line in Figure 5b illustrates the required time delay distribution of the metamaterial lens to achieve a target focal length of 76 mm. As an example, considering the variation of *a* with an error standard deviation, *σ* = 0.01 mm, the resulting time delays are also shown in Figure 5b, where larger variations can be seen to be concentrated around the lens center. This is because that the unit cells around the lens center have a larger geometrical ratio and hence a greater time delay sensitivity to manufacturing errors, as shown in Figure 2. However, it can also be seen that for this example, the difference is greatest at *x* = ±10 mm. This is because *r* cannot exceed unity, and this leads to a saturating effect as shown in Figure 3a which reduces the delay variation around *x* = 0.

A Huygens’ principle model can be then applied to predict the resulting wave pressure field and assess the performance of the AMMLs. The details of Huygens’ principle model can be found in Section 1, Supporting Information. The modeling approach can be summarized as follows: 1) Target AMML design: the desired AMML structure can be determined using the precalculated time delay performance of the chosen unit cells and the ADM. The desired parameters of the unit cells can be determined using the data presented in Figure 2; 2) Time delay estimation of unit cells with manufacturing errors: considering each individual manufacturing error of the chosen unit cells with a normal distribution with zero mean and a standard deviation of *σ*, perform Monte Carlo simulations of the variations of parameters of unit cells. The achieved time delay distribution of unit cells can be determined from results such as Figure 3; 3) Overall time delay estimation of an AMML with manufacturing errors: calculate the resulting overall time delay of all AMMLs from step (2) using the ADM via Equation (1); and 4) Acoustic pressure field simulations from the resulting time delay from step (3): calculate the resulting wave pressure field using the Huygens’ principle model and use the resulting focal lengths and focal zones to assess the lens performance under manufacturing errors.

## 4 Lens Performance Assessment and Manufacturing Error Effects

### 4.1 Lens Performance Assessment

**Figure 6:**
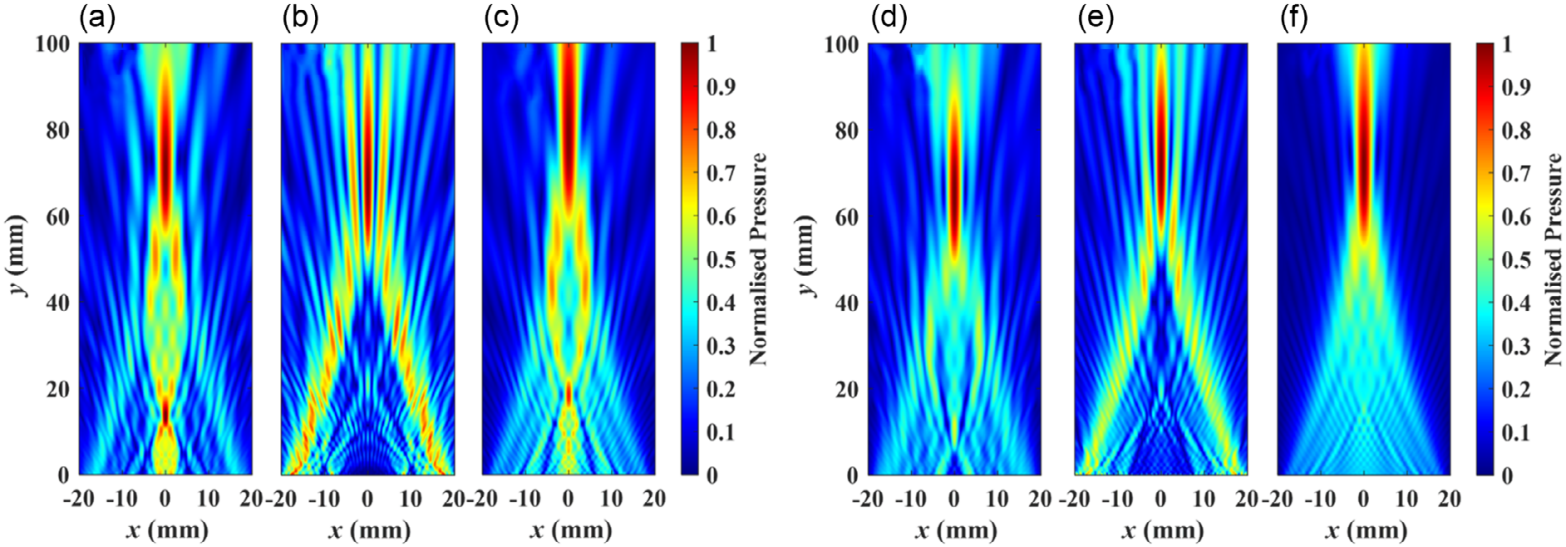
Normalized acoustic pressure field of the designed AMMLs constructed using: a–c) large unit cells for different unit cell types, i.e., 0.4, 0.27, and 0.49 mm, respectively, and d–f) small unit size of *s* = 0.15 mm. In the figure, a,d) are from the lenses constructed using steel cross unit cells, b,e) are from the lenses using resin circular void unit cells, and c,f) are from the lenses using silicone–resin layered unit cells.

Before assessing the lens performance with manufacturing errors, it is crucial to confirm that the designed metamaterial lenses can achieve the required focusing performance. To validate the performance of metamaterial lenses with different unit cells, the FE method (Comsol Multiphysics 6.1) is utilized for calculating the acoustical wave pressure fields, and the results are shown in **Figure** **6**. Details of the FE simulations are provided in the Experimental Section. According to the simulation results, all the designed lenses meet the specified requirements within an acceptable tolerance range. Lenses with smaller unit sizes demonstrate superior performance, including fewer side lobes, compared to those with larger units. However, fluctuations in focal positions can be attributed to the varying coupling effects and transmission characteristics of unit cells. Comparing the FE results with the predictions of Huygens’ principle model reveals a good agreement between them (the results are shown in the Section 2, Supporting Information). Therefore, the following section will discuss the effects of manufacturing errors on lens performance using the predictions of Huygens’ principle model with ADM: different unit cell structures with a unit size of *s* = 0.15 mm in the homogenous regime; and the silicone–resin layered unit cell with different unit sizes.

### 4.2 Manufacturing Error Effects on Lens Performance

**Figure 7:**
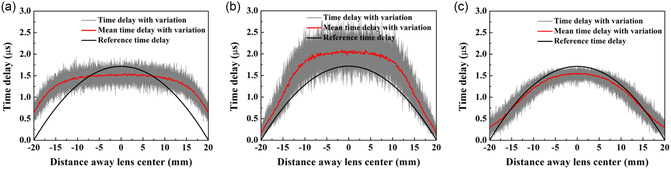
Time delay distribution comparison of AMMLs constructed using: a) steel cross unit cells; b) resin circular void unit cells; and c) silicone–resin layered unit cells. Note that, in each figure, the black line denotes the desired time delay distribution, the gray curves represent the actual time delay distributions with manufacturing errors from 100 realizations, while the red line indicates the average time delay distribution derived from these realizations. The used unit size is *s* = 0.15 mm and the standard deviation of dominant manufacturing error (Δ*a*, Δ*d*, and Δ*t*) is *σ* = 0.05 mm.

The acoustic pressure field of lenses with manufacturing errors is calculated using a Huygens’ principle model based on the resulting time delay distribution that accounts for these errors in the unit cells. An illustrative comparison of the resulting time delays of AMMLs constructed with various unit cell types with and without manufacturing errors is presented in **Figure** **7**. Using a unit size of *s* = 0.15 mm and considering a standard deviation of manufacturing error of *σ* = 0.05 mm, it is shown that AMMLs with silicone–resin layered unit cells are more robust to the manufacturing errors than the others. From Figure 7a it can be seen that the mean achieved time delay distribution of the steel cross unit cells does not follow the desired distribution and results in an imperfectly focused beam. This is caused by the asymmetry of gradient of the design curves shown in Figure 2 and 3, which means that positive and negative manufacturing errors have differing magnitudes of effect on the time delay. Hence, the mean delay over many realizations of the manufacturing errors is not equal to the target time delay. Figure 7b shows that, like the previous example, the mean achieved time delay distribution of the lens with resin circular void unit cells also does not follow the desired distribution, meaning that the correct focusing is again not achieved. This distortion also arises due to the asymmetry of the gradient of the design curves. From Figure 7c, for the lens constructed with silicone–resin layered unit cells, the mean achieved time delays of the lens again does not meet the required delay distribution, but the distortion is less than seen for the steel cross and resin circular void unit cells. This is because the variation of delay with geometry ratio for the silicone–resin layered unit cells (i.e., Figure 2) is closer to linear and this means that negative and positive manufacturing errors have similar and opposite effects. Hence, the mean, when taken over many realizations, is close to the target value.

**Figure 8:**
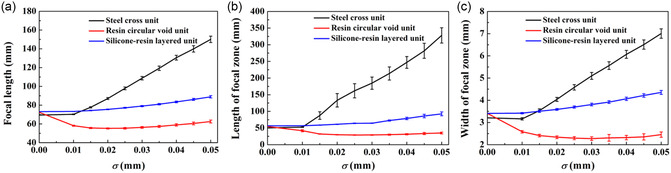
The performance of AMMLs as a function of the standard deviation of dominant manufacturing error, indicated by the resulting: a) focal length; b) length of focal zone; and c) width of focal zone. Note that the error bars represent the standard deviation derived from 100 realizations for each manufacturing error.

The performance of AMMLs with manufacturing errors at various standard deviation levels is assessed using parameters extracted from the resulting wave pressure fields, including the focal length and the focal spot size. The focal spot size is defined by the size of the focal zone with an amplitude of −6 dB relative to the peak amplitude and indicated by the length of the focal zone (in the *y* direction) and the width of the focal zone (in the *x* direction). **Figure** **8**a–c compares these parameters for AMMLs constructed using various unit cell types. The results demonstrate that AMMLs with steel cross units are 4.7–5.1 times more sensitive to manufacturing errors than the other unit types, as evidenced by the variation range of focal lengths. Similarly, the length and width of the focal spot exhibit the same trend. Both the focal length and focal spot size of the metamaterial lens with steel cross units exhibit an increasing trend with the increasing manufacturing errors. As discussed above, this is caused by the achieved mean time delay not matching the desired time delay, i.e., Figure 7a, hence leading to a distortion in the focusing performance. The performance of lenses with resin circular void unit cells initially shows a slight decrease in focal length, followed by an increase as manufacturing errors increase; however, the overall trend is one of decline in performance. This is again due to the distorting effect of the gradient of the design curves, i.e., Figure 7b. By contrast, the performance of lenses with silicone–resin layered unit cells is less sensitive to manufacturing errors, due to the less distorting effect as shown in Figure 7c.

**Figure 9:**
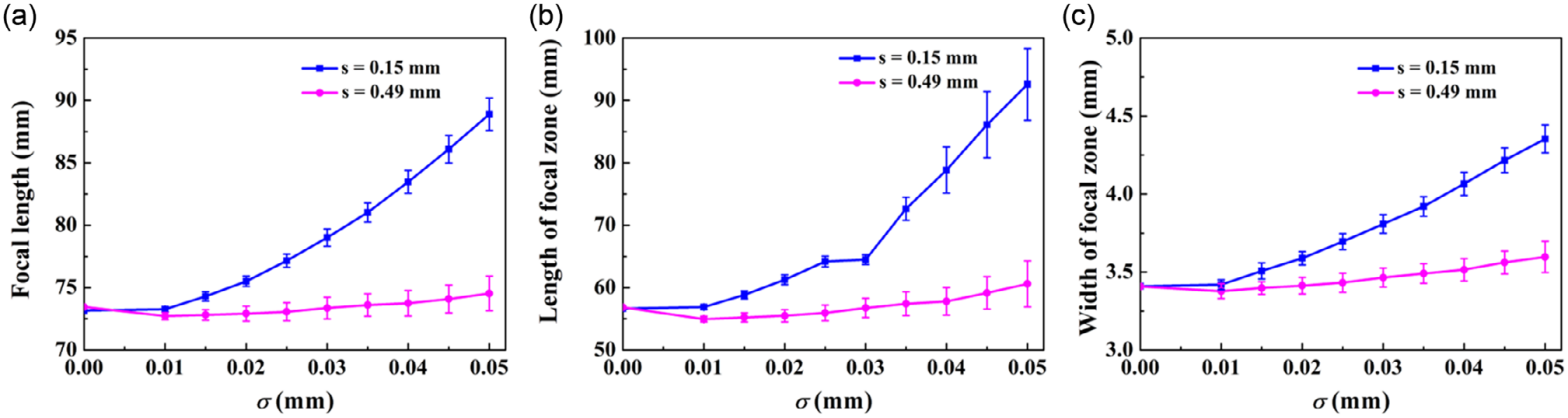
The performance of AMMLs constructed using silicone–resin layered unit cells as a function of the standard deviation of silicone thickness error Δ*t*, indicated by the resulting: a) focal length; b) length of focal zone; and c) width of focal zone. Note that the error bars represent the standard deviation derived from 100 realizations for each manufacturing error.

To examine the effects of unit size on the performance of AMMLs with manufacturing errors, we use the lens with silicone–resin layered units shown in Figure 1c as an example, chosen as it has been found to perform well in the presence of manufacturing errors. The performance characteristics are depicted in **Figure** **9**a–c. For both larger and smaller unit cells, the overall trend is reduced performance with increased manufacturing error; however, the performance of the larger unit cells initially exhibits a slight apparent improvement, for example, a reduced focal zone size, before the performance decreases for larger manufacturing errors. This is because the desired time delay profile is distorted by the manufacturing error, meaning that the actual time delay at the lens center is reduced, leading to a slightly shorter focal length, which then has a smaller focal zone size. When comparing the lens performance across different unit sizes, the performance of the lens with larger unit cells remains stable, whereas the performance of the lens with smaller unit cells is much more sensitive to manufacturing errors. This is because a given manufacturing error has a proportionately larger effect on a smaller unit cell. It can also be seen that the variance in performance increases with manufacturing error.

**Figure 10:**
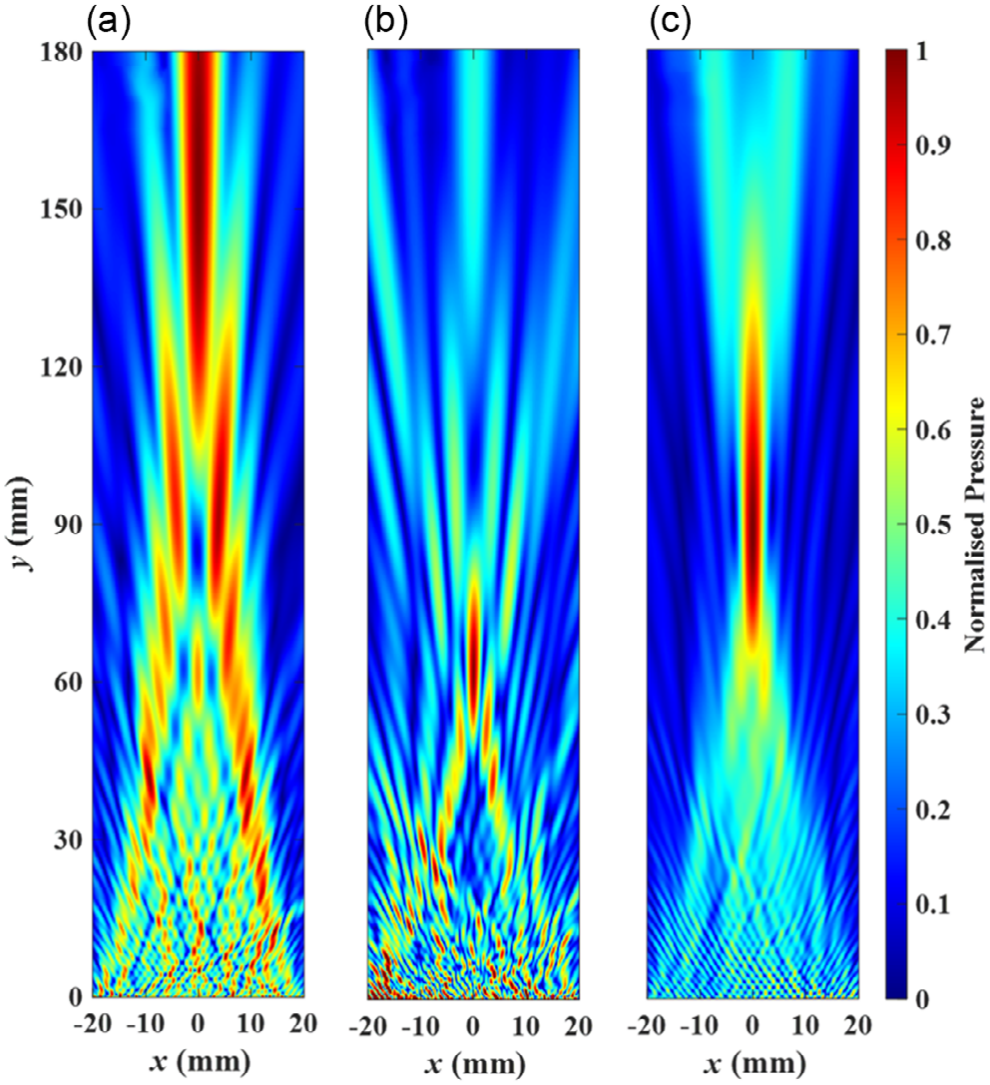
Normalized acoustic pressure field calculated using Huygens’ principle model from the designed AMMLs constructed using the unit size of *s* = 0.15 mm for different unit cell types with dominant manufacturing errors (standard deviation *σ* = 0.05 mm). In this figure, a) is from the lens constructed using steel cross unit cells, b) is from the lens using resin circular void unit cells, and c) is from the lens using silicone–resin layered unit cells.

To evaluate the performance of AMMLs constructed from various unit cell types under manufacturing errors, **Figure** **10** presents examples of normalized acoustic fields predicted using Huygens’ principle model. In these scenarios, the unit size is set to *s* = 0.15 mm and the standard deviation of dominant manufacturing error is *σ* = 0.05 mm. Based on the comparison between Figure 10a–c, the acoustic pressure field of the lens with silicone–resin layered unit cells demonstrates superior performance compared to the other unit cells. Notably, its focal length is significantly more stable and aligns more closely with the target requirements compared to the other two unit cell types. Comparing these results with those presented in Figure S1d–f, Section 2, Supporting Information, which exclude manufacturing errors, clearly demonstrates the impact of manufacturing errors on the performance of AMMLs.

## 5 Conclusion

This article explores the performance of AMMLs with manufacturing errors. During the design procedure, the achieved time delay distribution of AMMLs can be calculated using the ADM while considering the manufacturing errors for individual unit cells. Then, a Huygens’ principle model is implemented to predict the lens performance, including the focal length and dimensions of the focal spot. This is advantageous for precisely controlling the performance of a designed metamaterial lens. The results shown in Figure 8, 9 can serve as a route to designing metamaterial lenses using these types of unit cells. For an example, scenario shown in Figure 10, it is shown that the acoustic field and stability of focal performances of the metamaterial lens composed of silicone–resin layered units outperform the other two cases.

The results presented in Section 4 can be summarized into a simple criterion for assessing suitable fabrication methods. Selecting the appropriate manufacturing method depends on the acceptable tolerance of the metamaterial lens performance and critical indicators such as focal length and focal spot size. We use the silicone–resin layered unit as an example, with the focal length of the lens chosen as the main indicator. For instance, if the lens focal length of 76 ± 10 mm at a frequency of 1 MHz is set as the acceptable tolerance, this makes the maximum acceptable standard deviation of silicone thickness error Δ*t* for the silicone–resin layered unit *σ* = 0.04 mm, and the maximum acceptable absolute manufacturing error of the unit, i.e., 3*σ*, is 0.12 mm. According to the manufacturing resolution of Vipers, ProJets, and iPros 3D printers, a 3D printing method called stereolithography with normal resolution can be selected for lens fabrication, having tolerances of ±0.1 mm for well‐designed parts.

## 6 Experimental Section

6.1

6.1.1

##### Numerical Simulations

To assess the performance of metamaterial lenses with different unit cells and unit sizes, the FE method is employed to predict the resulting acoustical fields. The simulations were performed using commercial FE software (COMSOL Multiphysics 6.1). Figure S2, Section 3, Supporting Information shows an example schematic of the simulation model of the metamaterial lens with steel cross unit cells. The simulation was conducted in the frequency domain pressure acoustic module. In the simulation, the mass density and sound speed of the background medium (water) are *ρ*_0_ = 1000 kg m^−3^ and *c*_0_ = 1500 m s^−1^, respectively. The mass density and sound speed of the steel are *ρ*_steel_ = 7890 kg m^−3^ and *c*_steel_ = 5900 m s^−1^, respectively. The model has surrounding perfectly matched layers around the lens structures to prevent reflections from these boundaries, and a 1 MHz sinusoidal pressure of 1 Pa was applied to the bottom boundary of the lens.

For the metamaterial lenses utilizing resin circular void unit cells, the simulation model is identical to that used in the example case. However, the frequency domain acoustic–solid interaction module is utilized for the lens with resin circular void unit cells, and a prescribed sinusoidal displacement was applied to the bottom boundary of the lens. The mass density and sound speed of the resin are *ρ*_resin_ = 1300 kg m^−3^ and *c*_resin_ = 2400 m s^−1^, respectively.^[^ ^35^ ^]^ For the simulation of the lens with silicone–resin layered unit cells, the same model and boundary conditions as those used for the lens with steel cross unit cells are employed. The following material properties are used: the mass density and sound speed of the silicone are *ρ*_silicone_ = 1264 kg m^−3^ and *c*_silicone_ = 980 m s^−1^, respectively, while the mass density and sound speed of the resin are *ρ*_resin1_ = 1180 kg m^−3^ and *c*_resin1_ = 2700 m s^−1^, respectively.^[^ ^1^ ^]^

## Conflict of Interest

The authors declare no conflict of interest.

## Author Contributions

**Feng Qin**: validation (lead); writing—original draft (lead). **Jie Zhang**: supervision (equal); writing—review & editing (equal). **Bruce W. Drinkwater**: supervision (equal); writing—review & editing (equal).

## Supporting information

Supplementary Material

## Data Availability

The data that support the findings of this study are available from the corresponding author upon reasonable request.
