# Supplementary material for: The Effects of Manufacturing Errors on the Performance of Acoustic Metamaterial Lenses Operating in the MHz Regime
Source: Small Sci. 2024 Dec 6;5(4):2400481. doi: 10.1002/smsc.202400481 (PMC12244514; doi:10.1002/smsc.202400481)
Supplement: Supplementary file 1 — Supplementary Material [file SMSC-5-2400481-s001.pdf]

Supporting Information

The effects of manufacturing errors on the performance of acoustic metamaterial lenses operating in the MHz regime

Feng Qin, Jie Zhang and Bruce W. Drinkwater*

Department of Mechanical Engineering, University Walk, University of Bristol, Bristol, BS8 1TR, UK
E-mail: feng.qin@bristol.ac.uk

Section 1. Huygens' principle model

According to Huygens' principle, each point on an existing wavefront acts as a point source, emitting spherical wavelets. The envelope of these wavelets forms a new spherical wavefront, which in turn gives rise to a subsequent generation of spherical wavelets. This is mathematically explained as the superposition of fields from point sources. In this paper, the Huygens' principle model is utilized for predicting the acoustic field of an AMML:

$$P(x, y) = \sum_{n=1}^{n=N} \frac{1}{\sqrt{d_n}} e^{i(kd_n - \omega\tau_n)} ,$$

where, P is the acoustic pressure at (x, y), N is the number of unit cells at the lens' front, d_n is the distance between the nth unit cell and the point at (x, y), k is the wavenumber of the background material (water), defined as $k = 2\pi/\lambda_0$, ω is the angular frequency and $\omega = 2\pi f$, and τ_n is the time delay at the nth unit cell on the lens' front.

Section 2. Predicted acoustic field using the ADM and Huygens' principle model

The calculated pressure fields using the Huygens' principle model are shown in Figure S1. It is shown an excellent agreement between Figure 6 and Figure S1.

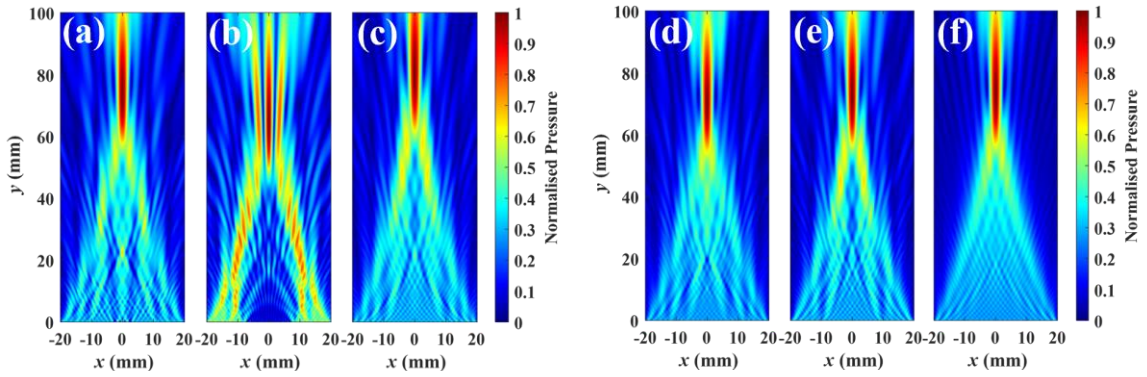

Figure S1. Normalized acoustic pressure field calculated using Huygens' principle model from the designed AMMLs constructed using: (a) (b) (c) large unit sizes for different unit cell types, i.e. 0.4 mm, 0.27 mm, and 0.49 mm, respectively; and (d) (e) (f) small unit size of s = 0.15 mm. In the figure, (a) (d) are from the lens constructed using steel cross unit cells, (b) (e) are from the lens using resin circular void unit cells, and (c) (f) are from the lens using silicone-resin layered unit cells.

Section 3. The FE model

Figure S2 shows an example schematic of the simulation model of the metamaterial lens with steel cross unit cells.

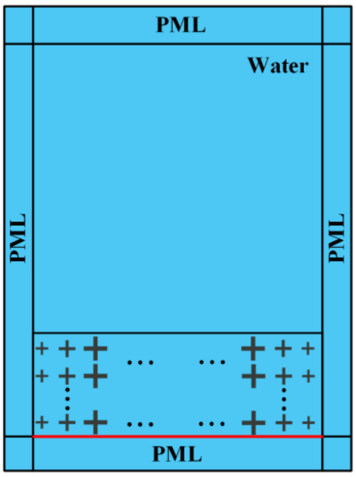

Figure S2. Schematic diagram of the FE model of the metamaterial lens consisting of steel cross unit cells with boundary conditions. PMLs are used around the simulation domain, and the red solid line on the bottom of the lens indicates the input plane wave.
